# Uncomplicated term vaginal delivery following magnetic resonance-guided focused ultrasound surgery for uterine fibroids

**DOI:** 10.2349/biij.6.2.e28

**Published:** 2010-04-01

**Authors:** S Zaher, D Lyons, L Regan

**Affiliations:** Department of Academic Obstetrics and Gynaecology, Imperial College, St. Mary’s Hospital, London, United Kingdom

**Keywords:** Magnetic resonance guided focused ultrasound surgery

## Abstract

A 35 year-old para 1+0 underwent MRgFUS per study protocol for multiple uterine fibroids, the largest of which measured 5 cm. She conceived 10 months following the procedure. The patient was induced at 41+6 weeks and underwent a normal vaginal delivery.

## INTRODUCTION

Magnetic Resonance-guided Focused Ultrasound Surgery (MRgFUS) was developed as a non-invasive alternative to conventional surgical techniques. It combines the use of high-powered ultrasound energy with Magnetic Resonance (MR) technology for accurate visualisation, control and feedback, allowing for safe thermo-ablation of tumours. The U.S. Food and Drug Administration (FDA) approved the technology in 2004. Data supporting the safety and efficacy of MRgFUS as an alternative treatment option for symptomatic uterine fibroids have been published previously [1, 2].

MRgFUS offers several advantages for treating uterine fibroids, in that it is a completely non-invasive, outpatient procedure that requires minimal sedation and allows for a speedy recovery. Patients undergoing MRgFUS typically return to work within 24 hours, compared with ten days after Uterine Artery Embolisation (UAE) and 6 weeks after myomectomy or hysterectomy. The initial FDA recommendation was that only women who had completed their families should be treated with MRgFUS. However, with the advantage of consistently good safety and efficacy results being reported, multi-centre fertility studies were commenced and are on-going. These studies are recruiting women with symptomatic uterine fibroids, who wish to become pregnant. The non-invasive nature of ExAblate, whereby only the uterine fibroids undergo thermal ablation with no damage to healthy surrounding tissue, suggests that MRgFUS should be a safe approach for women who want to preserve their fertility.

## CASE

A 39 year-old para 1+0 presented to the fibroid clinic at the authors’ hospital with a known diagnosis of fibroids made 3 years ago. The patient’s first pregnancy resulted in a premature delivery at 28 weeks (attributed to fibroids) of a 1.17kg female infant. She presented to the unit with a history of fibroid-related menorraghia and urinary pressure symptoms including frequency and nocturia. Symptoms were assessed using the Uterine fibroid-specific quality of life Questionnaire (UFS-QOL) [3]. The patient’s baseline score was 65. The severity of symptoms is directly related to the greatness of the score with a maximum score being 100.

In July 2006, a radiological MRI assessment deemed the patient to be suitable for treatment with MRgFUS. She was enrolled in an ongoing clinical trial of MRgFUS for treatment of symptomatic fibroids, where the subjects consisted of women wishing to preserve their fertility. The patient gave informed consent and fulfilled all eligibility criteria for the study.

**Figure 1:**
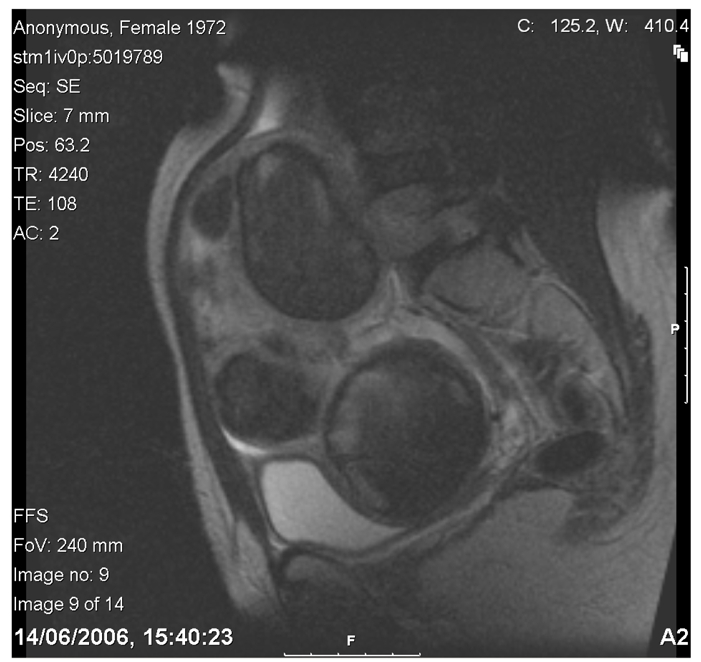
T2 weighted sagittal image prior to treatment. Maximum uterine dimension measuring 13.9cm, uterine volume of 509cc.

The patient’s screening MRI (see fig.1) revealed an enlarged fibroid uterus with an inferio-superior diameter of 13.5 cm. Five fibroids were identified, the largest being a fundal posterior intra-mural fibroid of 5 cm, as well as three other anterior intramural fibroids, and a low anterior wall fibroid of 4.5 cm which distorted the endometrial cavity posteriorly. All fibroids showed a hypo-intense signal as compared to myometrial tissue on T2-weighted imaging.

**Figure 2:**
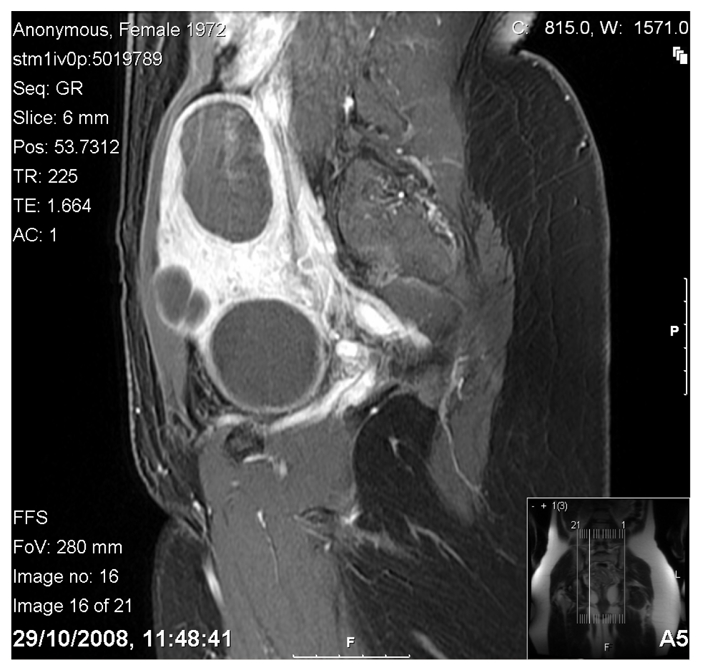
Contrast enhanced image showing non-perfusion of all fibroids, and overall shrinkage. Uterine volume now shrinking to 235cc.

The patient had a pre-treatment course of 3 GnRH analogue s/c injections, of which the first was given on the first day of the cycle and subsequent injections 28 days after the previous. She then underwent MRgFUS treatment in October 2006. Immediate post-treatment contrast-enhanced images revealed excellent results with approximately 90% non-perfusion of total fibroid volume achieved.

The treatment did not produce any complications and the patient was discharged on the same day. By 6 months post-procedure, the patient had experienced almost complete resolution of her pre-treatment symptoms, with her UFS-QOL score now 24. The patient conceived 4 months later, with no antenatal problems noted at booking. All booking observations were normal. An anomaly scan performed at 20 weeks gestation again revealed no abnormalities. Serial sonography at 28, 32, 36 and 40 weeks demonstrated appropriate foetal growth with cephalic presentation and an anterior placenta, which was not low-lying. There were no myomata viable on the 40-week scan.

At 41 +6 weeks the patient was admitted for induction of labour as she was past her due date. Labour was augmented by artificial rupture of the membranes and lasted for 5 hours 8 minutes. Foetal monitoring throughout was reassuring, and an uncomplicated vaginal delivery of a healthy female infant with a birth weight of 3580gms was achieved. Apgar scores were 8 at 1 minute, 9 at 5 minutes and 9 at 10 minutes. In the institution at which the patient delivered, cord gases are not routinely done for non-distressed infants. No paediatrician was required at delivery.

At 6 months post-delivery, the patient continued to maintain symptomatic improvement, with a QOL of 22. Contrast-enhanced MRI shows persistent non-perfusion of all fibroids; in addition, there has been a shrinkage in the overall uterine volume of 2.5 cm.

## DISCUSSION

The relationship between leiomyomas and infertility remains a subject for debate. The incidence of myomas in infertile women without any obvious cause of infertility is estimated to be between 1% and 2.4% [4]. However, there are no studies that compare pregnancy rates in women with and without fibroids, and the causal relationship seems to have been assumed from case studies of women who have conceived after their fibroids were removed [5].

A shift in cultural trends of women delaying pregnancy has resulted in many women presenting with symptomatic fibroids at a stage when preservation of the uterus is a priority. The National Office of Statistics in the United Kingdom reports a 74% increase in the number of conceptions in the 40-44 age group in 2004, when compared to that of 1988 [?]. This has resulted in a dilemma for the gynaecologist who is faced with providing an effective solution to his patient’s fibroid symptoms, while ensuring no detrimental effect on her fertility. The current standard of practice remains surgical in the many forms of myomectomy (laparotomy, laparoscopy, hysteroscopy).

Despite myomectomy being the gold standard, few studies agree on the actual increase in pregnancy rates following surgery, this being described as anything from 44%-81%; however, all report a fall in the rate of pregnancy loss [6-9].

Unfortunately fertility enhancement is not the only factor to be considered when recommending treatment; complications related to fibroid removal also have to be taken into account. During myomectomy, part of the uterine wall is severed in order to enucleate the fibroid. This damage to the wall is independent of the surgical technique used. Surgical sutures are placed in order to control bleeding and close the severed uterine wall, which results in a fibrotic scar. In addition to the resulting relative weakness of the uterine wall post-surgery – which may result in the low but major risk of uterine rupture during pregnancy/labour – adhesions can also form within the abdomen, resulting in mechanical infertility.

A further point to be considered is the risk of surgery-associated complications. Even if uncommon, intra-operative complications, such as bladder, bowel, ureteral injury, severe bleeding and unintended conversion to hysterectomy, have been reported. Moreover, post-operative complications, such as fistula or thrombosis and embolism, may also occur [10]. The minimally invasive treatment option of UAE has increased in popularity and reported pregnancies following treatment are plentiful. Although most pregnancies following UAE have good outcomes, the risk of pre-term delivery, spontaneous abortion, abnormal placentation and post-partum haemorrhage, are increased following uterine artery embolisation compared to myomectomy. This may be due to the resultant ischaemia, which not only occurs in the fibroid, but the entire uterus. This ischaemia may result in chronic weakness of the pregnant uterus. Uterine rupture has been reported with both myomectomy and UAE.

Although both myomectomy and UAE are seen as effective and safe treatment options for fibroids, the increase in pregnancy complications seen with UAE means that patients desiring future fertility should be recommended myomectomy as the treatment of choice over UAE.

With MRgFUS, heat ablation is limited to the core of the fibroid and no damage to the surrounding uterine wall occurs. Real-time monitoring of the volume of ablation enables limitation of the thermal damage to a distinct targeted region of the fibroid as shown in pathology specimens [11] and MR-contrast imaging. Accordingly, there arises the hypothesis that MRgFUS may enable non-invasive treatment of uterine fibroids in women desiring pregnancy, without compromise to the integrity of the uterus or increase in pregnancy-related risks.

This out-patient procedure with minimal sedation requirements and a speedy recovery time, allows patients to return to work within 24 hours, compared to 10 days after UAE and 6 weeks with myomectomy. Over 4000 women with symptomatic uterine fibroids have been treated worldwide. Published studies have shown that up to 84.6% of women treated experienced significant symptomatic improvement at 24 months post-treatment follow up [12]. However, initial FDA guidelines still recommend that only women who have completed their families undergo this treatment, despite there having been three published studies of achieved pregnancies following MRgFUS treatment [13-16].

This paper describes the first pregnancy and successful delivery in a woman who was part of a fertility trial and specifically treated with MRgFUS for symptomatic fibroids that had caused a previous premature delivery at 28 weeks.

Fertility data continues to accumulate and the results are encouraging. MRgFUS appears to be a safe fertility-preserving treatment option for fibroids.
